# Supplementary material for: Comparative effectiveness of surgery in traumatic acute subdural and intracerebral haematoma: study protocol for a prospective observational study within CENTER-TBI and Net-QuRe
Source: BMJ Open. 2019 Oct 16;9(10):e033513. doi: 10.1136/bmjopen-2019-033513 (PMC6797419; doi:10.1136/bmjopen-2019-033513)
Supplement: Author's manuscript [file bmjopen-2019-033513.draft_revisions.pdf]

BMJ Open is committed to open peer review. As part of this commitment we make the peer review history of every article we publish publicly available.

When an article is published we post the peer reviewers' comments and the authors' responses online. We also post the versions of the paper that were used during peer review. These are the versions that the peer review comments apply to.

The versions of the paper that follow are the versions that were submitted during the peer review process. They are not the versions of record or the final published versions. They should not be cited or distributed as the published version of this manuscript.

BMJ Open is an open access journal and the full, final, typeset and author-corrected version of record of the manuscript is available on our site with no access controls, subscription charges or pay-per-view fees (http://bmjopen.bmj.com).

If you have any questions on BMJ Open's open peer review process please email info.bmjopen@bmj.com

BMJ Open

Comparative effectiveness of surgery in traumatic acute subdural and intracerebral hematoma: study protocol for a prospective observational study within CENTER-TBI and Net-QuRe

Journal:	*BMJ Open*
Manuscript ID	bmjopen-2019-033513
Article Type:	Protocol
Date Submitted by the Author:	08-Aug-2019

Complete List of Authors:	Van Essen, Thomas; Leiden University Medical Centre, Haaglanden Medical Centre and Haga Teaching Hospital , University Neurosurgical Centre Holland; ErasmusMC - University Medical Centre Rotterdam, Centre for Medical Decision Sciences, Department of Public Health<br>Volovici, Victor; ErasmusMC - University Medical Centre Rotterdam, Department of Neurosurgery; ErasmusMC - University Medical Centre Rotterdam, Centre for Medical Decision Sciences, Department of Public Health,<br>Cnossen, MC; ErasmusMC - University Medical Centre Rotterdam, Centre for Medical Decision Sciences, Department of Public Health<br>Kolias, Angelos; Addenbrooke's Hospital, University of Cambridge, Department of Neurosurgery; University of Cambridge, NIHR Global Health Research Group on Neurotrauma<br>Ceyisakar, Iris; ErasmusMC - University Medical Centre Rotterdam, Centre for Medical Decision Sciences, Department of Public Health<br>Nieboer, Daan; ErasmusMC - University Medical Centre Rotterdam, Centre for Medical Decision Sciences, Department of Public Health<br>Peppel, Lianne; Rijndam Rehabilitation, ErasmusMC - University Medical Center Rotterdam, Department of Rehabilitation Medicine<br>Heijenbrok-Kal, Majanka; Rijndam Rehabilitation, ErasmusMC - University Medical Center Rotterdam, Department of Rehabilitation Medicine<br>Ribbers, Gerard; Rijndam Rehabilitation, ErasmusMC - University Medical Center Rotterdam, Department of Rehabilitation<br>Menon, David; Addenbrooke's Hospital, University of Cambridge, Division of Anaesthesia<br>Hutchinson, Peter; Addenbrooke's Hospital, University of Cambridge, Department of Neurosurgery; University of Cambridge, NIHR Global Health Research Group on Neurotrauma<br>Depreitere, Bart; University Hospital KU Leuven, Department of Neurosurgery<br>de Ruiter, Godard; Leiden University Medical Centre, Haaglanden Medical Centre and Haga Teaching Hospital , University Neurosurgical Centre Holland<br>Lingsma, Hester; ErasmusMC - University Medical Centre Rotterdam, Centre for Medical Decision Sciences, Department of Public Health<br>Steyerberg, Ewout; ErasmusMC - University Medical Centre Rotterdam, Centre for Medical Decision Sciences, Department of Public Health, ;

Leiden University Medical Centre and Haaglanden Medical Centre, Department of Biomedical Data Sciences
Maas, Andrew; Antwerp University Hospital and University of Antwerp, Department of Neurosurgery
Peul, Wilco; Leiden University Medical Centre, Haaglanden Medical Centre and Haga Teaching Hospital , University Neurosurgical Centre Holland

Keywords: NEUROSURGERY, EPIDEMIOLOGY, Traumatic brain injury, Acute subdural hematoma, Intracerebral hematoma

SCHOLARONE™ Manuscripts

Comparative effectiveness of surgery in traumatic acute subdural and intracerebral hematoma: study protocol for a prospective observational study within CENTER-TBI and Net-QuRe

Thomas A. van Essen (corresponding author)[1,2]

Victor Volovici[2,3]

Maryse Cnossen[2]

Angelos Kolias[4,5]

Iris Ceyisakar[2]

Daan Nieboer[2]

Lianne D. Peppel[6]

Majanka H. Heijenbrok-Kal[6]

Gerard M. Ribbers[6]

David K. Menon[7]

Peter Hutchinson[4,5]

Bart Depreitere[7]

Godard C.W. de Ruiter[1]

Hester F. Lingsma[2]

Ewout W. Steyerberg[2,8]

Andrew I.R. Maas[9]

Wilco C. Peul[1]

Affiliations:

[1] University Neurosurgical Centre Holland, Leiden University Medical Centre, Haaglanden Medical Centre and Haga Teaching Hospital, Leiden and The Hague, Albinusdreef 2, 2333 ZA Leiden, The Netherlands

[2] Centre for Medical Decision Sciences, Department of Public Health, ErasmusMC - University Medical Center Rotterdam, Rotterdam, The Netherlands

For peer review only

[3] Department of Neurosurgery, ErasmusMC - University Medical Center Rotterdam, Rotterdam, The Netherlands

[4] Division of Neurosurgery, Department of Clinical Neurosciences, University of Cambridge and Addenbrooke's Hospital, Cambridge, United Kingdom

[5] NIHR Global Health Research Group on Neurotrauma, University of Cambridge, Cambridge, United Kingdom

[6] Rijndam Rehabilitation and Department of Rehabilitation Medicine, ErasmusMC - University Medical Center Rotterdam, The Netherlands

[7] Division of Anaesthesia, University of Cambridge and Addenbrooke's Hospital, Cambridge, United Kingdom

[8] Department of Neurosurgery, University Hospital Leuven, Leuven, Belgium

[9] Department of Biomedical Data Sciences, Leiden University Medical Centre and Haaglanden Medical Centre, Leiden and The Hague, The Netherlands

[10] Department of Neurosurgery, Antwerp University Hospital, Edegem, and University of Antwerp, Belgium

<u>Corresponding author:</u> Thomas A. van Essen

Address: University Neurosurgical Centre Holland, Leiden University Medical Centre, Haaglanden Medical Centre and Haga Teaching Hospital, Leiden and The Hague, Albinusdreef 2, 2333 ZA Leiden, The Netherlands

For peer review only

Centre for Medical Decision Sciences, Department of Public Health, ErasmusMC - University Medical Center Rotterdam, Rotterdam, The Netherlands

Telephone: 003171 526 2109

Fax: 003171 526 6987

e-mail address: t.a.vanessen@erasmusmc.nl

Wordcount: abstract 292, manuscript 3980

Number of figures: 1

For peer review only

For peer review only - http://bmjopen.bmj.com/site/about/guidelines.xhtml

Abstract

Introduction

Controversy exists about the optimal treatment for patients with a traumatic acute subdural hematoma (ASDH) and an intracerebral hematoma/contusion (t-ICH). Treatment varies largely between different regions. The effect of this practice variation on patient outcome is unknown.

Here we present the protocol for a prospective multicentre observational study aimed at comparing the effectiveness of different treatment strategies in patients with ASDH and/or t-ICH. Specifically, the aims are to compare 1) an acute surgical approach to an expectant approach and 2) craniotomy to decompressive craniectomy when evacuating the hematoma.

Methods and analysis

Patients presenting to the emergency room with an ASDH and/or an t-ICH are eligible for inclusion. Standardized prospective data on patient and injury characteristics, treatment, and outcome will be collected on 1000 ASDH and 750 t-ICH patients in 60-70 centres within two multicentre prospective observational cohort studies: the Collaborative European NeuroTrauma Effectiveness Research in Traumatic Brain Injury (CENTER-TBI) and Neurotraumatology Quality Registry (Net-QuRe). The interventions of interest are acute surgery, defined as surgery directly after the first CT at presentation, versus late or no surgery, and craniotomy versus decompressive craniectomy. The primary outcome measure is the Glasgow Outcome Score – Extended at six months. Secondary outcome measures include in-hospital mortality, quality of life and neuropsychological tests. In the primary analysis, the effect of treatment preference (e.g. proportion of patients in which the intervention under study is preferred) per hospital will be analysed with random effects ordinal regression models, adjusted for case-mix and stratified by study. Such a hospital level approach reduces confounding by indication. Sensitivity analyses will include propensity score matching, with treatment defined on patient level. This study is designed to determine the best acute management strategy for ASDH and t-ICH by exploiting the existing between-hospital variability in surgical management.

Ethics and dissemination

For peer review only

Ethics approval was obtained in all participating countries. Results of surgical management of acute subdural hematoma and intracerebral hematoma/contusion will separately be submitted for publication in a peer-reviewed journal.

Trial registration

CENTER-TBI is registered within ClinicalTrials.gov with identifier NCT02210221 and Net-QuRe is registered with the Netherlands Trial Register NL 5761.

Article summary

Strengths and limitations of this study

- This comparative effectiveness study is a multicentre prospective observational cohort study that exploits variation in management strategies for intracranial hematomas to enable comparisons of the effectiveness of interventions.
- To overcome the bias by confounding the main analyses uses an instrumental variable approach; this approach is more robust to address unmeasured confounding than conventional individual patients level analysis methods
- Large sample sizes will be recruited: 1000 patients with acute subdural hematoma and 750 patients with intracerebral hematoma/contusion are expected, recruited in approximately 70 centres.
- Simulation studies confirmed the expected samples to be sufficient.
- The main limitation of this study is the absence of a randomised assignment of treatment strategy.

Keywords

Acute subdural hematoma, traumatic contusion, intracerebral hematoma, decompressive craniectomy.

For peer review only

Background

In Europe, over two million patients are admitted to hospital each year for traumatic brain injury (TBI), of whom 82.000 people die [1]. Survivors may have long-term physical, cognitive and mental disorders that often necessitate specialized care or rehabilitation programs. This debilitating morbidity has been estimated to lead to enormous societal costs [2]. An acute intracranial hematoma is the most frequently encountered pathological entity in TBI patients (Figure). Predominantly two specific subtypes, the acute subdural and an intracerebral hematoma or contusion (ASDH and t-ICH), occur in respectively 11 and 8% of all moderate TBI patients and up to 49% and 35% of all severe TBI patients [3-6]. Patients with an ASDH and/or t-ICH can show a wide array of symptoms, ranging from relatively mild complaints such as headache and nausea to severe conditions such as a comatose state (defined by a Glasgow Coma Scale (GCS) < 9).

Management of these traumatic hematomas can be challenging and requires the integration of clinical findings and diagnostic imaging. The main question is whether or not the patient needs to be immediately operated upon for evacuation of the hematoma and, secondly, if this surgical evacuation should be accompanied by a decompressive craniectomy (DC, i.e. leaving the bone flap out) or not. Among comatose patients with a large ASDH, direct evacuation of the hematoma leads to a lower mortality [3,4]. Although questioning the effectiveness of surgery in these patients has been compared to questioning the effectiveness of a parachute in skydiving [5,6], this cannot be generalized to most ASDH patients. Surgery may save a patient’s life and preserve neurological function, some, however, may have an unsatisfactory functional outcome, ranging from severe neurological and cognitive deficits to a persistent vegetative state [7-9]. Furthermore, certain subgroups may not benefit from surgery because the damage by the primary injury is simply too devastating [9]. On the other hand, surgery may not always be necessary and a substantial proportion of patients managed conservatively have satisfactory outcomes [10-14]. In addition, timing of surgery plays a role, specifically for a t-ICH. Sometimes a t-ICH is initially managed conservatively, but may later be treated surgically when a patient deteriorates. The evacuation of the t-ICH can consist of removal of contused brain tissue. Finally, for the decision whether or not the evacuation of the hematoma should be accompanied by a DC, the surgeon weighs the increased complications rate of a DC against the risk of medically intractable diffuse brain swelling [15].

For peer review only

High quality evidence for these decisions (*if*, *when* and *how)* is not available. For all guidelines that relate to TBI, the Brain Trauma Foundation (BTF) guideline for surgical management of intracranial hematomas, devised in 2005 by an international panel of experts, is based solely on class III evidence [16,17]. As a result these complex decisions are often based on intuition, regional training and experience of the surgeon, leading to broad practice variation between centres, countries and even between surgeons within a centre [5,18-22]

Therefore, a systematic evaluation in a (prospective) comparative study is proposed with comprehensive assessment of outcome, including perceived quality of life. We consider an observational comparative effectiveness design the next best alternative to a randomised controlled trial (RCT). These clinical questions are difficult to address in a randomised trial due to several methodological, ethical and pragmatic concerns [23]. Most importantly, the hesitance of clinicians to randomise surgical treatments because of strong opinions on the best treatment hampers realising a RCT.

Here, we present the design of a pragmatic prospective observational cohort study of surgical strategies for ASDH and t-ICH, conducted in the Collaborative European NeuroTrauma Effectiveness Research in Traumatic Brain Injury (CENTER-TBI) study and the Dutch embedded complete chain of care Neurotraumatology Quality Registry (Net-QuRe) [24,25].

Objectives

The primary aim is to compare the effectiveness of acute surgery with expectant management in the treatment of 1) ASDH and 2) t-ICH. The secondary goal is 3) to assess the effectiveness of craniotomy compared to DC for ASDH and/or t-ICH.

For peer review only

For peer review only - http://bmjopen.bmj.com/site/about/guidelines.xhtml

Methods and design

Design

This study uses a comparative effectiveness research (CER) design, a multicentre prospective observational cohort study that exploits variation in neurotrauma care to create and compare parallel study groups. The multicentre design is necessary to ensure the required number of patients with different neurotrauma treatment strategies for ASDH and t-ICH. The study is conducted in neurosurgical trauma centres in Europe that participate in CENTER-TBI [24]. CENTER-TBI collects data of patients with clinical diagnosis of TBI and an indication for a CT scan [26]. Data for the cohort described in this protocol will partly be collected through CENTER-TBI. The Dutch centres not participating in CENTER-TBI will acquire data through Net-QuRe, in a separate database with a similar data collection protocol [25,27]. The research question and methodology described here were designed before the inclusion of patients.

Patient and public involvement

Patients were involved in the priority of the research questions and selection of outcome measures in a 'patient advisory panel' consisting of patients and their caregivers. Furthermore, some patients and their caregivers have been asked to join a focus group on the feasibility of the follow-up (burden of the follow-up, the design/length of the questionnaires) and to advice on other research questions. The patient panel and their caregivers will be informed about the developments of the study and will be invited to participate in research meetings and discussions. Also, patients and family are informed of the study results through dedicated websites (Center-tbi.eu and Net-QuRe.nl).

A public debate is going on in the Netherlands about whether or not patients are treated too much at an older age or end-of-life stage. This debate has led to the start of formal campaign 'Choosing Wisely' of which the senior author WPE is organizing member [28]. The appropriateness of surgical TBI treatment has been prioritized.

Eligible study patients

The patients are selected from the observational cohorts from CENTER-TBI or Net-QuRe. Patients presenting to the emergency room with a clinical and radiological diagnosis of ASDH and/or a t-ICH

For peer review only

are eligible for inclusion.

The in- and exclusion criteria are as following.

Inclusion criteria:

- ASDH and/or a large (>10 cc) t-ICH on a CT-scan
- Acute presentation (< 24 hours of injury) with history of head trauma
- Clinical indication for admission (ward or ICU)

Exclusion criteria:

- An ASDH or t-ICH due to penetrating injury, a spontaneous or iatrogenic ASDH/t-ICH
- Severe pre-existing neurological disorder that would confound outcome assessments

Patients are not excluded based on other clinical and radiological characteristics (such as: advanced age, antiplatelet/anticoagulant use, small hematoma volume). Radiological criteria for an ASDH or t-ICH/contusion are a high-density lesion, with or without radiological signs of raised intracranial pressure (ICP) and with or without mass effect (i.e. midline shift, compression of ventricles and/or basal systems). The minimal size of the t-ICH needs to be 10cc to be included.

<u>Intervention procedures</u>

Inherent to the observational design of this study the management strategies under investigation proceed according to local emergency- and intensive care protocols or surgeon's expertise. Consequently, the resulting variation in management is accepted and analysed. To gain insight into this variation detailed information is collected on the reasons for specific interventions or management strategies (see section 'why' questions).

Surgical strategy

Surgical treatment consists of evacuation of the hematoma and/or contusion with a craniotomy, or with a DC, defined as hematoma evacuation plus leaving a large portion of the skull open to allow brain swelling in the secondary phase, preventing subsequent brain injury. Generally, in Europe a craniotomy is performed for hematoma evacuation and DC when (intractable) swelling is seen intraoperatively or when swelling is expected (preventive). The decision for a DC can be made primarily, or secondarily by increasing the defect of the bone flap that is formed during a normal

For peer review only - http://bmjopen.bmj.com/site/about/guidelines.xhtml

craniotomy. In conjunction to these surgical procedures the neurosurgeon will decide to place an intracranial pressure (ICP) monitor or not. The ICP device can be an intraparenchymal sensor or an external ventricular drain with a transducer for the ICP. The latter has an option to drain cerebrospinal fluid (CSF) and thereby lower ICP.

The operation will be performed by a qualified neurosurgeon or neurosurgical resident. The techniques for evacuating such hematomas are well established, although specific components of the operations may differ between surgeons. Our aim will be to collect pertinent operative data on a standardized data collection form.

The postoperative care on the ward or intensive care unit (ICU) generally is protocolized in European centres. The length of hospital stay will differ considerably between patients, ranging from one day to several months, mainly related to the severity of the injury. The aim for a patient is to be discharged as soon as possible. Furthermore, the supportive care is provided as described in the section 'expectant management'.

Expectant management, with possibly delayed surgery

For patients admitted to the ward, monitoring is in general by clinical neurological control (GCS and motor strength) with or without CT brain follow-up, whereas for those patients admitted to the ICU (mostly severe TBI) the diagnostic and therapeutic options include ICP monitoring with medical management of intracranial hypertension (i.e. hyperosmolar therapies, hyperventilation etc) and cerebral perfusion pressure (CPP).

Follow-up CT scans, performed during hospitalization, are collected and analysed centrally, independent from the treating physicians. Hereby, an estimation is made about the proportion of the evacuated hematoma and the change in density of lesions.

Patients will be allocated to one of the treatment arms based on the initial treatment strategy. The data collection includes questions after each CT that ask whether or not the patient is transferred to the OR for an operative procedure. In doing so, the treatment 'arms' in this study can be carefully controlled based on the first CT on presentation (showing an ASDH and/or a t-ICH). The initial treatment regimen chosen will be one of either treatment arms, analysed according to an intention-to-treat approach.

For peer review only - http://bmjopen.bmj.com/site/about/guidelines.xhtml

Data collection

Data of care management by hospital personnel are registered in all departments. Data collection is done in a standardized electronic database, based on the 'common data elements' for TBI and web-based data collection protocol [29,30].

Practice variation and provider profiling

Parallel to a survey on regional practice variation towards ASDH management in the Netherlands, we have performed provider profiling of neurosurgical care for TBI in Europe [31]. This study provides an exploration of the organization of neurosurgical care and treatment policy of TBI in all study centres. Such centre characterization allows specification of the local policy and standardized protocol. Structures and processes of care to be studied are patient volume, location of first ER evaluation, level of the trauma centre, referral policy, number of neurosurgeons, type of ICU and 24/7 CT availability. In addition, with regard to post-acute care, routine follow-up for ER patients, ICU approaches to fluid load, hyperventilation, hyperosmolar therapy, timing of intracranial surgery, timing of extracranial surgery, glucose management, cerebral spinal fluid (CSF) drainage, DC, CPP management, coagulopathy treatment and ICP monitoring (parenchymal or CSF catheter). Whether or not consensus for divergent clinical decisions is agreed upon and whether or not hospital protocols are available and applied.

Admission data

In short, the 'common data elements' entail the collection of patient characteristics including demographics, comorbidities, associated extracranial injuries, neurological condition, prehospital information, hypoxic and hypotensive periods, and CT abnormalities. For the purpose of CER detailed information on processes of care will be collected within CENTER-TBI and Net-QuRe, including timing (of first CT, second CT, operation), surgery parameters, pre-hospital management and IC therapies (including ICP: pharmacological and/or DC). These data are collected on patient level, but also on hospital level (see previous paragraph).

For peer review only

'Why' questions

The observational design poses a challenge for inferring that the surgery caused the outcome instead of other factors. Several known and measured confounders (pupillary abnormalities, GCS and hematoma characteristics on CT) can be accounted for. However, for the decision to operate or not in intracranial hematoma, neurosurgeons select certain clinical, radiological and subjective 'gut feeling' characteristics that would normally go unmeasured. Therefore, the following efforts are undertaken to collect variables that normally will go unmeasured. To assess the effectiveness of different treatments for ASDH and t-ICH additional data are collected on an individual (doctor) level; the neurosurgeon, ICU physician and/or neurologist is asked to give their indication/reason to choose for surgery or conservative management (e.g. hematoma size, mass effect, clinical symptoms, clinical deterioration and/or other motivation), his or her motivation for the chosen procedure (DC or craniotomy) and the anticipated prognosis of the patient.

The motivation for surgery and the prognosis is collected before the decision for surgery or conservative management because afterwards the motivation could have been changed due to several factors such as intraoperative findings and the clinical course of the patient after the surgery. Because of the infrastructure in TBI care, in which the clinician on-call is often outside the hospital and - in case of the neurosurgeon - will start with the surgery right after arrival in the hospital, the neurological and neurosurgical residents will assess their supervising clinician's motivation before the decision is carried out. To control this process, the date and time of collecting these variables is collected as well.

For peer review only

<u>Outcome measurements and endpoints</u>

Within CENTER-TBI and Net-QuRe the outcome measures are assessed by face-to-face interviews, postal or emailed questionnaires or by telephone interviews at 3, 6 and 12 months after injury. Outcome assessment is done naïve for the research questions.

The primary endpoint is the 6-month Glasgow Outcome Score - Extended (GOSE, [32]). The Glasgow Outcome Scale Extended (GOSE) is the most commonly used outcome measure in TBI. The GOSE grades disability on an 8-point scale incorporating physical deficits as well as emotional

and cognitive disturbances affecting disability [33,34]. The GOSE is designed as a structured interview and can be applied through telephone [35] and the mail [36].

Secondary endpoints are mortality, structural hematoma changes on CT, frequency and type of neurosurgical interventions, ICU and hospital length of stay (days), complications (hydrocephalus, intracranial haemorrhage, infection, pulmonary embolism, deep vein thrombosis and death), 'treatment failure' during the initial hospital admittance (patient in the expectant group who are operated at a delayed moment or patients in the early surgery group who are operated again), discharge to home (from the hospital, rehabilitation facility or nursing home), quality of life 6 and 12 months postinjury with the SF-12, the brain injury specific Quality of Life after Brain Injury questionnaire (Qolibri) [37] and cognitive tests. Details of these and other outcome measures are provided in our previous publication [24].

<u>Data analysis</u>

Patient characteristics, hospital characteristics, and variation in both treatment and outcome variation will be described using descriptive statistics. To assess differences between groups, appropriate tests will be employed according to distribution and scale of measurement (Student's t-tests or Mann-Whitney U tests for continuous variables and Chi-squared tests, or Fisher's exact test for categorical variables). To examine effectiveness of interventions, proportional odds logistic regression models with the 8-point ordinal GOSE as outcome variable will be used. A proportional odds model increases statistical power in comparison to a conventional logistic regression model with a binary outcome [38]. The odds ratios derived from a proportional odds regression model could be interpreted as the odds ratio (OR) for shifting over the GOSE [38].

The main challenge in the analyses is how to estimate a treatment effect in these observational data with strong confounding. Conventional methods, patient-level analyses with covariate adjustment in regression modelling and propensity score matching, can insufficiently account for the (unmeasured) confounding in TBI [39]. Therefore, the main analyses will use the between-hospital variation in treatment for determining effectiveness by comparing regional treatment strategies. This is an instrumental variable (IV) approach. The instrument is the proportion of patients exposed to the

For peer review only

intervention per hospital, determined as the proportion initially operated ASDH of total ASDH patients, initially operated t-ICH of total t-ICH patients and proportion of total ASDH or t-ICH patients exposed to primary DC (that forms a proxy for 'aggressiveness' of the neurosurgical staff). Total ASDH and ICH numbers are available through the registry. The instrument is entered as an independent variable to the analyses. The unmeasured and measured confounding at the hospital level, for example hospitals that perform more surgery also more often perform other treatments, is overcome with a multilevel analysis model [40]. In this model the random intercept should capture the measured and unmeasured confounders at hospital level, resulting in unbiased treatment effect estimates. The random intercept for each hospital represents the unexplained hospital effect (beyond all factors included in the model, including the instrument treatment preference). Assumptions of the IV approach will be checked according to our previous published case study [39].

For these analyses, hospitals contributing at least 15 patients to the study sample are included to minimize the influence of chance. To increase statistical power, adjustment for potential patient level confounding will be made by adding the strongest predictors of outcome (age, GCS, pupillary response, CT characteristics (hematoma thickness and volume, subarachnoid haemorrhage, basal cistern compromise, other focal or diffuse lesions), hypoxic or hypotensive episodes and extracranial injuries) as covariates [41]. These factors were determined by prognostic modelling in the International Mission for Prognosis and Analysis of Clinical Trials (IMPACT) study based on a dataset of ten RCTs and three observational studies [42]. Thus, the treatment effect parameter will be the estimate for the effect of 'aggressiveness' on outcome from a random effects ordinal regression model with hospital as a random intercept.

In sensitivity analyses the instrument validity will be further explored by quantifying a priori collected data, the results of our survey [18] and the provider profiling of CENTER-TBI [19], and comparing these to the post-hoc derived relative proportion exposed to the intervention per hospital.

In the secondary analyses, conventional regression modelling with covariate adjustment and propensity score matching is performed. In both these analyses actual treatment will be a binary treatment variable and GOSE as ordinal outcome variable. For ASDH surgery effectiveness,

For peer review only

confounding will be controlled for by adding age, GCS, pupil reactivity, hematoma thickness and midline shift as covariates in the model. For t-ICH effectiveness, confounding adjustment for age, GCS, hematoma volume, pupil reactivity, hematoma laterality and midline shift. For DC effectiveness, by age, GCS, pupil reactivity, midline shift and hematoma size (ASDH: thickness; t-ICH: volume).

Importantly, the effect of surgery is probably not uniform, as is suggested by empirical evidence [43,44] and by clinical experience. Therefore, effect modification by the following variables will be tested using interaction terms: GCS of ≥ 9, hematoma size >10 mm in diameter, midline shift >5 mm, time to treatment and baseline prognostic risk [32].

<u>Sample size</u>

In CENTER-TBI and Net-QuRe together approximately 1000 patients with ASDH are expected. For t-ICH, 750 patients are expected. Parallel to the core study, 3500 and 3000 ASDH and ICH patients are expected in the registry respectively. These patients are recruited in approximately 70 centres. Standard sample size calculations for these specific analyses are not readily available. Therefore, a simulation study was performed to calculate statistical power. The assumptions for these calculations were the following: 30, 50 or 70 hospitals, variation of intracranial operation among hospitals 10 to 90% and an effect estimate of OR 0.6 on unfavourable outcome. In addition, we assumed covariate adjustment and the ordinal analysis to increase power with 40 to 49% [38]. The simulation confirmed these sample sizes to be sufficient to obtain a power of 80% to detect a difference (assuming a 2-sided significance 0.05).

This is in line with preparatory simulation study we performed in which a true treatment effect was simulated specifically to assess the TBI specific associations between covariates and outcome [39]. This simulation study was built around the International and North American Tirilazad trial dataset (86 hospitals between 1992 and 1994) of the IMPACT dataset [42], which was inflated to CENTER-TBI/Net-QuRe numbers (respectively 750 and 1000 patients from 70 hospitals). We simulated a hypothetical intervention with an OR 1.5. For the association between the hypothetical intervention and confounders, we used the observed associations between intracranial operation and confounders in the Tirilazad dataset. We used six-month binary (functional) outcome as dependent variable, which

For peer review only

was generated based on a combination of the prognostic effect of the confounders and the effect of the hypothetical intervention.

For analyses and simulations R statistical software with add-ons (the *rms* and *lme4* packages) is used.

<u>Missing data</u>

Missing baseline data will be imputed with multiple imputation (n=5).

<u>Reporting</u>

Reporting of our study will follow the STROBE statement with a special focus on instrumental variable analyses recommendations [45]

<u>Study limitations</u>

The main limitation of this study is the absence of a randomised assignment of treatment or strategy. Risks of confounding by indication are reduced by instrumental variable analysis. The success of the primary analysis however, depends on the strength of the instrument, i.e. the difference in aggressive versus conservative practice style between physicians during the study period. The results of the provider profiling (before the study) are encouraging. As secondary analyses we perform more conventional approaches to adjust for confounding by indication (multivariable adjustment and propensity scores). The results of analytical approaches will be interpreted in the light of the assumptions they require and to what extent these are likely to be fulfilled in the data. The final conclusion will be drawn based on the joint results of all analyses.

For peer review only

For peer review only - http://bmjopen.bmj.com/site/about/guidelines.xhtml

Discussion

There is controversy with regard to the initial neurosurgical management of ASDH and t-ICH. First, neurosurgeons are faced with an acute decision to operate or not, and second, are confronted by the choice to evacuate the hematoma with or without a DC. The complexity lies in the balance between too liberal surgical indications with an increased number of survivors with severe disabilities against inappropriate conservative management with unnecessary death and disability. In combination with the circumstances, i.e. urgency and time pressure as well as absence of peer consultation, these treatment decisions have been shown to lead to variation in surgical treatment between surgeons.

The proposed study will provide a strong level of evidence for surgical management of ASDH and t-ICH. We expect that the large natural existing practice variation in management of these intracranial lesions [18,19] could in part explain the unexplained between-centre variability in outcome in TBI [46]. Thereby, the impact on patients will probably be significant. Recognizing and implementing the most effective clinical treatment strategy could be an important step towards reducing the widely differing injury mortality rates across Europe [47].

Current and ongoing studies are sparse. Since the BTF guideline, which was based on merely retrospective studies with small or selected study populations that were performed more than 10 years before the guideline [5,10,11,48], there have been only some comparative studies. In our own retrospective analyses, early ASDH evacuation might be associated with lower odds of mortality and unfavourable outcome (GOS $\leqq$ 3) [43]. This is the first report showing an effect estimate of surgery for ASDH. The clinical effectiveness of an early evacuation for t-ICH was challenged in the Surgical Trial in Traumatic Intracerebral Haemorrhage (STITCH-Trauma), an international multicentre pragmatic randomised controlled trial [49]. The study started in October 2009 but was halted due to a disbalance in recruited patients per country. In the analysis of the included patients, a strong (but non-significant) tendency towards benefit of early surgery was found on the primary endpoint the dichotomous GOS and there were significantly more deaths in the initial conservative treatment group. The effectiveness of a primary DC in patients with ASDH is currently being assessed in the recruiting RESCUE-ASDH randomised trial [15].

For peer review only - http://bmjopen.bmj.com/site/about/guidelines.xhtml

Thus, these traumatic hematomas confront the neurosurgeon with a challenging surgical decision-making task, which, most likely due to a lack of general evidence, leads to broad variation in current surgical practice patterns. While RCTs can be delivered and provide high level evidence, they are challenging to conduct, hence the rationale for other methodological intervention paradigms. The high-quality observational study presented in this article, with a focus on the analysis of the differences in management and outcome, is expected to provide the much-needed further evidence in the field of surgical management of traumatic focal lesions.

For peer review only

For peer review only - http://bmjopen.bmj.com/site/about/guidelines.xhtml

Abbreviations

ASDH: acute subdural hematoma,

CENTER-TBI: Collaborative European NeuroTrauma Effectiveness research in TBI

CER: comparative effectiveness research,

CPP: cerebral perfusion pressure,

CSF: cerebrospinal fluid,

CT: computed tomography,

ER: emergency room,

GCS: Glasgow Coma Score,

GOSE: Glasgow Outcome Scale Extended,

ICU: intensive care unit,

ICP: intracranial pressure,

IMPACT: International Mission for Prognosis and Analysis of Clinical Trials in TBI,

IV: instrumental variable,

Net-QuRe: Neurotraumatology Quality Registry,

OR: operating room,

Qolibri: Quality of Live in Brain Injury,

RCT: randomised clinical trial,

TBI: traumatic brain injury,

t-ICH: intracerebral hematoma/contusion.

For peer review only

Declarations

Ethics and dissemination

Ethical approval for the studies CENTER-TBI and Net-QuRe in the Netherlands was obtained from the committee on medical ethics ('Commissie Medische Ethiek') from Leiden University Medical Centre. Appropriate local ethical approval is sought from each participating centre in the study before recruitment is started. Written informed consent is obtained from the patient or the legal first representative on the ER and preferably as soon as possible. Center-TBI is registered at ClinicalTrials.gov (NCT02210221) and Net-QuRe is registered at the Netherlands Trial Register (NL 5761) [26,27].

Results of surgical management of ASDH and t-ICH will separately be submitted for publication in a peer-reviewed journal.

The data that support the findings of this study are available but restrictions apply to the availability of these data, which were used under license for the current study, and so are not publicly available. Data are however available from the authors upon reasonable request, with permission of the CENTER-TBI and Net-QuRe management teams.

Competing interests

The authors declare that they have no competing interests.

Funding

The project described in this study protocol is funded by the *Hersenstichting Nederland* (the Dutch Brian Foundation, grant number ps2014.06) for Net-QuRe and the European Union seventh Framework Program (grant 602150) for CENTER-TBI. Additional support for CENTER-TBI was obtained from the Hannelore Kohl Stiftung (Germany), from OneMind (USA), from Integra LifeSciences Corporation (USA), and from NeuroTrauma Sciences (USA). The funding bodies had no role in the design of the study, collection, analysis, and interpretation of data and in writing the manuscript. This research received no specific grant from any funding agency in the public, commercial or not-for-profit sectors.

For peer review only

Authors PH and AK are supported by the NIHR Global Health Research Group on Neurotrauma, which was commissioned by the National Institute for Health Research (NIHR) using UK aid from the UK Government (project 16/137/105). The views expressed in this publication are those of the author(s) and not necessarily those of the NIHR or the Department of Health and Social Care. PH is supported by a Research Professorship from the NIHR, the NIHR Cambridge Biomedical Research Centre, and the Royal College of Surgeons of England. AK is supported by the Royal College of Surgeons of England and a Clinical Lectureship, School of Clinical Medicine, University of Cambridge

Author contributions

TvE conceived of the study design, performed the statistical analysis and wrote the manuscript. GdR, VV, MC, LP, HL, BD, MH, PH, AK, ES and WP participated in the design of the study and helped to draft the manuscript. DN, IC, HL and TvE performed the simulation studies. All authors read and approved the final manuscript.

Acknowledgements

Not applicable

For peer review only

References

1 Majdan M, Plancikova D, Brazinova A, *et al.* Epidemiology of traumatic brain injuries in Europe: a cross-sectional analysis. *Lancet Public Health* 2016;**1**:e76–e83. doi:10.1016/S2468-2667(16)30017-2

2 Faul M, Wald MM, Rutland-Brown W, *et al.* Using a Cost-Benefit Analysis to Estimate Outcomes of a Clinical Treatment Guideline: Testing the Brain Trauma Foundation Guidelines for the Treatment of Severe Traumatic Brain Injury. *The Journal of Trauma: Injury, Infection, and Critical Care* 2007;**63**:1271–8. doi:10.1097/TA.0b013e3181493080

3 Seelig JM, Becker DP, Miller JD, *et al.* Traumatic acute subdural hematoma: major mortality reduction in comatose patients treated within four hours. *N Engl J Med* 1981;**304**:1511–8. doi:10.1056/NEJM198106183042503

4 Bullock MR, Chesnut R, Ghajar J, *et al.* Surgical management of acute subdural hematomas. *Neurosurgery* 2006;**58**:S16–24–discussionSi–iv.

5 Compagnone C, Murray GD, Teasdale GM, *et al.* The Management of Patients with Intradural Post-Traumatic Mass Lesions: A Multicenter Survey of Current Approaches to Surgical Management in 729 Patients Coordinated by the European Brain Injury Consortium. *Neurosurgery* 2005;:1183–92. doi:10.1227/01.NEU.0000186239.10915.09

6 Smith GCS, Pell JP. Parachute use to prevent death and major trauma related to gravitational challenge: systematic review of randomised controlled trials. *BMJ* 2003;**327**:1459–61.

7 Tallon JM, Ackroyd-Stolarz S, Karim SA, *et al.* The epidemiology of surgically treated acute subdural and epidural hematomas in patients with head injuries: a population-based study. *Can J Surg* 2008;**51**:339–45.

8 Li LM, Kolias AG, Guilfoyle MR, *et al.* Outcome following evacuation of acute subdural haematomas: a comparison of craniotomy with decompressive craniectomy. *Acta Neurochir* 2012;**154**:1555–61. doi:10.1007/s00701-012-1428-8

9 Nijboer JMM, van der Naalt J, Duis ten H-J. Patients beyond salvation? Various categories of trauma patients with a minimal Glasgow Coma Score. *Injury* 2010;**41**:52–7. doi:10.1016/j.injury.2009.05.030

10 Dent DL, Croce MA, Menke PG, *et al.* Prognostic factors after acute subdural hematoma. *The Journal of Trauma: Injury, Infection, and Critical Care* 1995;**39**:36–42–discussion42–3.

11 Mathew P, Oluoch-Olunya DL, Condon BR, *et al.* Acute subdural haematoma in the conscious patient: outcome with initial non-operative management. *Acta Neurochir* 1993;**121**:100–8.

12 Bus S, Verbaan D, Kerklaan BJ, *et al.* Do older patients with acute or subacute subdural hematoma benefit from surgery? *Br J Neurosurg* 2019;**33**:51–7. doi:10.1080/02688697.2018.1522418

13 Gernsback JE, Kolcun JPG, Richardson AM, *et al.* Patientem Fortuna Adiuvat: The Delayed Treatment of Surgical Acute Subdural Hematomas-A Case Series. *World Neurosurg* 2018;**120**:e414–20. doi:10.1016/j.wneu.2018.08.095

14 Servadei F, Nasi MT, Cremonini AM, *et al.* Importance of a reliable admission Glasgow Coma Scale score for determining the need for evacuation of posttraumatic subdural hematomas: a prospective study of 65 patients. *The Journal of Trauma: Injury, Infection, and Critical Care* 1998;**44**:868–73.

For peer review only - http://bmjopen.bmj.com/site/about/guidelines.xhtml

15 Kolias AG, Viaroli E, Rubiano AM, *et al.* The Current Status of Decompressive Craniectomy in Traumatic Brain Injury. *Current Trauma Reports* 2018;:1–7. doi:10.1007/s40719-018-0147-x

16 Bullock MR, Chesnut R, Ghajar J, *et al.* Surgical management of acute subdural hematomas. *Neurosurgery* 2006;**58**:S16–24–discussionSi–iv.

17 Bullock MR, Chesnut R, Ghajar J, *et al.* Surgical management of traumatic parenchymal lesions. *Neurosurgery* 2006;**58**:S25–46–discussionSi–iv.

18 van Essen TA, de Ruiter GCW, Kho KH, *et al.* Neurosurgical Treatment Variation of Traumatic Brain Injury: Evaluation of Acute Subdural Hematoma Management in Belgium and The Netherlands. *Journal of Neurotrauma* 2017;**34**:881–9. doi:10.1089/neu.2016.4495

19 Essen TA, Boogert HF, Cnossen MC, *et al.* Variation in neurosurgical management of traumatic brain injury: a survey in 68 centers participating in the CENTER-TBI study. 2018;:1–15. doi:10.1007/s00701-018-3761-z

20 Kolias AG, Scotton WJ, Belli A, *et al.* Surgical management of acute subdural haematomas: current practice patterns in the United Kingdom and the Republic of Ireland. *Br J Neurosurg* 2013;**27**:330–3. doi:10.3109/02688697.2013.779365

21 Ghajar J, Hariri RJ, Narayan RK, *et al.* Survey of critical care management of comatose, head-injured patients in the United States. *Crit Care Med* 1995;**23**:560–7.

22 Murray GD, Teasdale GM, Braakman R, *et al.* The European Brain Injury Consortium survey of head injuries. *Acta Neurochir* 1999;**141**:223–36.

23 Stein SC. Comparative effectiveness in neurosurgery: what it means, how it is measured, and why it matters. *Neurosurgical FOCUS* 2012;**33**:E1. doi:10.3171/2012.2.FOCUS1232

24 Maas AIR, Menon DK, Steyerberg EW, *et al.* Collaborative European NeuroTrauma Effectiveness Research in Traumatic Brain Injury (CENTER-TBI): a prospective longitudinal observational study. *Neurosurgery* 2015;**76**:67–80.

25 Van Essen TA, Peppel L, Van Dijck J, Dijkman M, Hester F. Lingsma, et al. The Net-QuRe study: an observational cohort Study on neurosurgical and rehabilitation strategies for traumatic brain injury. The 3rd Joint Symposium of the International and National Neurotrauma Societies and AANS/CNS Section on Neurotrauma and Critical Care August 11–16, 2018 Toronto, Canada. *Journal of Neurotrauma* 2018;**35**:A–1–A–285. doi:10.1089/neu.2018.29013.abstracts

26 Maas AIR. CENTER-TBI: Collaborative European NeuroTrauma Effectiveness Research in TBI. *clinicaltrialsgov* 2014.

27 Nederlands Trial Register [Internet]. Amsterdam: Academic Medical Center (The Netherlands). 2004 Oct 26. Identifier Trial NL5761; 2016 July 28. Available from: https://www.trialregister.nl/trial/5761.

28 Choosing Wisely Netherlands Campaign. https://www.demedischspecialist.nl/onderwerp/campagne-verstandig-kiezen.

29 Maas AIR, Harrison-Felix CL, Menon D, *et al.* Standardizing data collection in traumatic brain injury. *Journal of Neurotrauma* 2011;**28**:177–87. doi:10.1089/neu.2010.1617

30 Maas AI, Harrison-Felix CL, Menon D, *et al.* Common data elements for traumatic brain injury: recommendations from the interagency working group on demographics and

For peer review only

For peer review only - http://bmjopen.bmj.com/site/about/guidelines.xhtml

clinical assessment. *Arch Phys Med Rehabil* 2010;**91**:1641–9. doi:10.1016/j.apmr.2010.07.232

31 van Essen TA, Boogert den HF, Cnossen MC, *et al.* Variation in neurosurgical management of traumatic brain injury: a survey in 68 centers participating in the CENTER-TBI study. *Acta Neurochir* Published Online First: December 2018. doi:10.1007/s00701-018-3761-z

32 Wilson JT, Pettigrew LE, Teasdale GM. Structured interviews for the Glasgow Outcome Scale and the extended Glasgow Outcome Scale: guidelines for their use. *Journal of Neurotrauma* 1998;**15**:573–85.

33 Shukla D, Devi BI, Agrawal A. Outcome measures for traumatic brain injury. *Clin Neurol Neurosurg* 2011;**113**:435–41. doi:10.1016/j.clineuro.2011.02.013

34 Bullock MR, Merchant RE, Choi SC, *et al.* Outcome measures for clinical trials in neurotrauma. *Neurosurgical FOCUS* 2002;**13**:ECP1.

35 Pettigrew LEL, Wilson JTL, Teasdale GM. Reliability of ratings on the Glasgow Outcome Scales from in-person and telephone structured interviews. *Journal of Head Trauma Rehabilitation* 2003;**18**:252–8.

36 Wilson JTL, Edwards P, Fiddes H, *et al.* Reliability of postal questionnaires for the Glasgow Outcome Scale. *Journal of Neurotrauma* 2002;**19**:999–1005. doi:10.1089/089771502760341910

37 Steinbuechel von N, Petersen C, Bullinger M. Assessment of health-related quality of life in persons after traumatic brain injury--development of the Qolibri, a specific measure. *Acta Neurochir Suppl* 2005;**93**:43–9.

38 Roozenbeek B, Lingsma HF, Perel P, *et al.* The added value of ordinal analysis in clinical trials: an example in traumatic brain injury. *Critical Care* 2011;**15**:R127. doi:10.1186/cc10240

39 Cnossen MC, van Essen TA, Ceyisakar IE, *et al.* Adjusting for confounding by indication in observational studies: a case study in traumatic brain injury. *Clinical Epidemiology* 2018;**10**:841–52.

40 Li B, Lingsma HF, Steyerberg EW, *et al.* Logistic random effects regression models: a comparison of statistical packages for binary and ordinal outcomes. *BMC Med Res Methodol* 2011;**11**:77. doi:10.1186/1471-2288-11-77

41 Hernandez AV, Steyerberg EW, Butcher I, *et al.* Adjustment for strong predictors of outcome in traumatic brain injury trials: 25% reduction in sample size requirements in the IMPACT study. *Journal of Neurotrauma* 2006;**23**:1295–303. doi:10.1089/neu.2006.23.1295

42 Lingsma H, Andriessen TMJC, Haitsema I, *et al.* Prognosis in moderate and severe traumatic brain injury: external validation of the IMPACT models and the role of extracranial injuries. *J Trauma Acute Care Surg* 2013;**74**:639–46.

43 Van Essen TA, Dijkman M, Cnossen MC, *et al.* Comparative effectiveness of surgery for traumatic acute subdural hematoma in an aging population. *Journal of Neurotrauma* Published Online First: September 2018. doi:10.1089/neu.2018.5869

44 Gregson BA, Mitchell P, Mendelow AD. Surgical Decision Making in Brain Hemorrhage. *Stroke* 2019;**50**:1108–15. doi:10.1161/STROKEAHA.118.022694

For peer review only - http://bmjopen.bmj.com/site/about/guidelines.xhtml

45 Davies NM, Smith GD, Windmeijer F, *et al.* Issues in the reporting and conduct of instrumental variable studies: a systematic review. *Epidemiology* 2013;**24**:363–9. doi:10.1097/EDE.0b013e31828abafb

46 Lingsma HF, Roozenbeek B, Li B, *et al.* Large Between-Center Differences in Outcome After Moderate and Severe Traumatic Brain Injury in the International Mission on Prognosis and Clinical Trial Design in Traumatic Brain Injury (IMPACT) Study. *Neurosurgery* 2011;**68**:601–8. doi:10.1227/NEU.0b013e318209333b

47 Eurosafe. Injuries in the European Union: Summary of Injury Statistics for the Years 2008-2010. Amsterdam, The Netherlands: European Association for Injury Prevention and Safety Promotion. Available at: http://ec.europa.eu/health/data_collection/docs/idb_report_2013_en.pdf. 2013.

48 Servadei F, Nasi MT, Cremonini AM, *et al.* Importance of a reliable admission Glasgow Coma Scale score for determining the need for evacuation of posttraumatic subdural hematomas: a prospective study of 65 patients. *The Journal of Trauma: Injury, Infection, and Critical Care* 1998;**44**:868–73.

49 Mendelow AD, Gregson BA, Rowan EN, *et al.* Early surgery versus initial conservative treatment in patients with traumatic intracerebral haemorrhage [STITCH(Trauma)]: the first randomised trial. *Journal of Neurotrauma* Published Online First: March 2015. doi:10.1089/neu.2014.3644

50 Maas AIR, Menon DK, Adelson PD, *et al.* Traumatic brain injury: integrated approaches to improve prevention, clinical care, and research. *The Lancet Neurology* 2017;**16**:987–1048. doi:10.1016/S1474-4422(17)30371-X

For peer review only

702 **Figure legend**

703

704 Figure: Different types of post-traumatic intracranial haematoma

705 (A) Epidural haematoma: a collection of blood between the skull and the outer membrane covering the
706 brain (dura mater). (B) Subdural haematoma: a collection of blood located underneath the dura mater,
707 generally associated with bruising of the underlying brain tissue (contusions). (C) Haemorrhagic
708 contusion and intracerebral haematoma: lesions that reflect similar underlying pathologies, ranging
709 from local bruising (contusions) to bleeding into the brain tissue (haematoma). Figure courtesy of
710 Maartje Kunen, Medical Visuals, Arnhem, Netherlands. Reproduced with permission from [50].

For peer review only

For peer review only - http://bmjopen.bmj.com/site/about/guidelines.xhtml

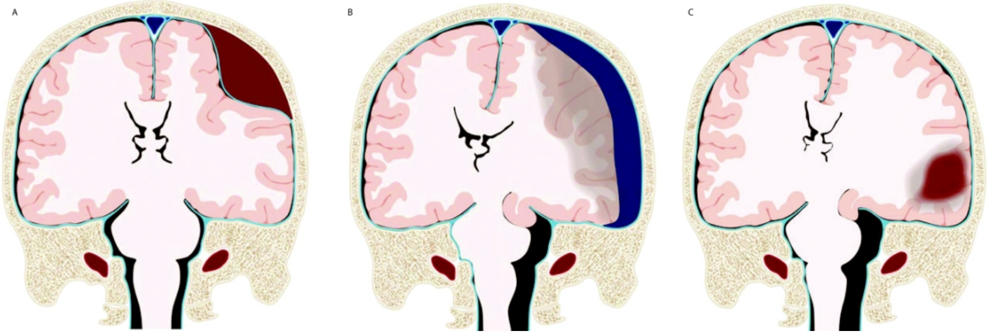

Figure: Different types of post-traumatic intracranial haematoma
(A) Epidural haematoma: a collection of blood between the skull and the outer membrane covering the brain (dura mater). (B) Subdural haematoma: a collection of blood located underneath the dura mater, generally associated with bruising of the underlying brain tissue (contusions). (C) Haemorrhagic contusion and intracerebral haematoma: lesions that reflect similar underlying pathologies, ranging from local bruising (contusions) to bleeding into the brain tissue (haematoma). Figure courtesy of Maartje Kunen, Medical Visuals, Arnhem, Netherlands. Reproduced with permission from [50].

448x149mm (300 x 300 DPI)

BMJ Open

Comparative effectiveness of surgery in traumatic acute subdural and intracerebral hematoma: study protocol for a prospective observational study within CENTER-TBI and Net-QuRe

Journal:	*BMJ Open*
Manuscript ID	bmjopen-2019-033513.R1
Article Type:	Protocol
Date Submitted by the Author:	22-Sep-2019
Complete List of Authors:	Van Essen, Thomas; Leiden University Medical Centre, Haaglanden Medical Centre and Haga Teaching Hospital , University Neurosurgical Centre Holland; ErasmusMC - University Medical Centre Rotterdam, Centre for Medical Decision Sciences, Department of Public Health<br>Volovici, Victor; ErasmusMC - University Medical Centre Rotterdam, Department of Neurosurgery; ErasmusMC - University Medical Centre Rotterdam, Centre for Medical Decision Sciences, Department of Public Health,<br>Cnossen, Maryse; ErasmusMC - University Medical Centre Rotterdam, Centre for Medical Decision Sciences, Department of Public Health<br>Kolias, Angelos; Addenbrooke's Hospital, University of Cambridge, Department of Neurosurgery; University of Cambridge, NIHR Global Health Research Group on Neurotrauma<br>Ceyisakar, Iris; ErasmusMC - University Medical Centre Rotterdam, Centre for Medical Decision Sciences, Department of Public Health<br>Nieboer, Daan; ErasmusMC - University Medical Centre Rotterdam, Centre for Medical Decision Sciences, Department of Public Health<br>Peppel, Lianne; Rijndam Rehabilitation, ErasmusMC - University Medical Center Rotterdam, Department of Rehabilitation Medicine<br>Heijenbrok-Kal, Majanka; Rijndam Rehabilitation, ErasmusMC - University Medical Center Rotterdam, Department of Rehabilitation Medicine<br>Ribbers, Gerard; Rijndam Rehabilitation, ErasmusMC - University Medical Center Rotterdam, Department of Rehabilitation<br>Menon, David; Addenbrooke’s Hospital, University of Cambridge, Division of Anaesthesia<br>Hutchinson, Peter; Addenbrooke's Hospital, University of Cambridge, Department of Neurosurgery; University of Cambridge, NIHR Global Health Research Group on Neurotrauma<br>Depreitere, Bart; University Hospital KU Leuven, Department of Neurosurgery<br>de Ruiter, Godard; Leiden University Medical Centre, Haaglanden Medical Centre and Haga Teaching Hospital , University Neurosurgical Centre Holland<br>Lingsma, Hester; ErasmusMC - University Medical Centre Rotterdam, Centre for Medical Decision Sciences, Department of Public Health<br>Steyerberg, Ewout; ErasmusMC - University Medical Centre Rotterdam, Centre for Medical Decision Sciences, Department of Public Health, ;

	Leiden University Medical Centre and Haaglanden Medical Centre, Department of Biomedical Data Sciences<br>Maas, Andrew; Antwerp University Hospital and University of Antwerp, Department of Neurosurgery<br>Peul, Wilco; Leiden University Medical Centre, Haaglanden Medical Centre and Haga Teaching Hospital , University Neurosurgical Centre Holland
**Primary Subject Heading**:	Neurology
Secondary Subject Heading:	Surgery, Epidemiology, Evidence based practice
Keywords:	NEUROSURGERY, EPIDEMIOLOGY, Traumatic brain injury, Acute subdural hematoma, Intracerebral hematoma

Comparative effectiveness of surgery in traumatic acute subdural and intracerebral hematoma: study protocol for a prospective observational study within CENTER-TBI and Net-QuRe

Thomas A. van Essen (corresponding author)[1,2]

Victor Volovici[2,3]

Maryse C. Cnossen[2]

Angelos Kolias[4,5]

Iris Ceyisakar[2]

Daan Nieboer[2]

Lianne D. Peppel[6]

Majanka H. Heijenbrok-Kal[6]

Gerard M. Ribbers[6]

David K. Menon[7]

Peter Hutchinson[4,5]

Bart Depreitere[8]

Godard C.W. de Ruiter[1]

Hester F. Lingsma[2]

Ewout W. Steyerberg[2,9]

Andrew I. Maas[10]

Wilco C. Peul[1]

Affiliations:

[1] University Neurosurgical Centre Holland, Leiden University Medical Centre, Haaglanden Medical Centre and Haga Teaching Hospital, Leiden and The Hague, Albinusdreef 2, 2333 ZA Leiden, The Netherlands

[2] Centre for Medical Decision Sciences, Department of Public Health, ErasmusMC - University Medical Center Rotterdam, Rotterdam, The Netherlands

For peer review only - http://bmjopen.bmj.com/site/about/guidelines.xhtml

[3] Department of Neurosurgery, ErasmusMC - University Medical Center Rotterdam, Rotterdam, The Netherlands

[4] Division of Neurosurgery, Department of Clinical Neurosciences, University of Cambridge and Addenbrooke's Hospital, Cambridge, United Kingdom

[5] NIHR Global Health Research Group on Neurotrauma, University of Cambridge, Cambridge, United Kingdom

[6] Rijndam Rehabilitation and Department of Rehabilitation Medicine, ErasmusMC - University Medical Center Rotterdam, The Netherlands

[7] Division of Anaesthesia, University of Cambridge and Addenbrooke's Hospital, Cambridge, United Kingdom

[8] Department of Neurosurgery, University Hospital Leuven, Leuven, Belgium

[9] Department of Biomedical Data Sciences, Leiden University Medical Centre and Haaglanden Medical Centre, Leiden and The Hague, The Netherlands

[10] Department of Neurosurgery, Antwerp University Hospital, Edegem, and University of Antwerp, Belgium

Corresponding author: Thomas A. van Essen

Address: University Neurosurgical Centre Holland, Leiden University Medical Centre, Haaglanden Medical Centre and Haga Teaching Hospital, Leiden and The Hague, Albinusdreef 2, 2333 ZA Leiden, The Netherlands

For peer review only

Centre for Medical Decision Sciences, Department of Public Health, ErasmusMC - University Medical Center Rotterdam, Rotterdam, The Netherlands

Telephone: 003171 526 2109

Fax: 003171 526 6987

e-mail address: t.a.vanessen@erasmusmc.nl

Wordcount: abstract 292, manuscript 3980

Number of figures: 1

For peer review only

Abstract

Introduction

Controversy exists about the optimal treatment for patients with a traumatic acute subdural hematoma (ASDH) and an intracerebral hematoma/contusion (t-ICH). Treatment varies largely between different regions. The effect of this practice variation on patient outcome is unknown.

Here we present the protocol for a prospective multicentre observational study aimed at comparing the effectiveness of different treatment strategies in patients with ASDH and/or t-ICH. Specifically, the aims are to compare 1) an acute surgical approach to an expectant approach and 2) craniotomy to decompressive craniectomy when evacuating the hematoma.

Methods and analysis

Patients presenting to the emergency room with an ASDH and/or an t-ICH are eligible for inclusion. Standardized prospective data on patient and injury characteristics, treatment, and outcome will be collected on 1000 ASDH and 750 t-ICH patients in 60-70 centres within two multicentre prospective observational cohort studies: the Collaborative European NeuroTrauma Effectiveness Research in Traumatic Brain Injury (CENTER-TBI) and Neurotraumatology Quality Registry (Net-QuRe). The interventions of interest are acute surgery, defined as surgery directly after the first CT at presentation, versus late or no surgery, and craniotomy versus decompressive craniectomy. The primary outcome measure is the Glasgow Outcome Score – Extended at six months. Secondary outcome measures include in-hospital mortality, quality of life and neuropsychological tests. In the primary analysis, the effect of treatment preference (e.g. proportion of patients in which the intervention under study is preferred) per hospital will be analysed with random effects ordinal regression models, adjusted for case-mix and stratified by study. Such a hospital level approach reduces confounding by indication. Sensitivity analyses will include propensity score matching, with treatment defined on patient level. This study is designed to determine the best acute management strategy for ASDH and t-ICH by exploiting the existing between-hospital variability in surgical management.

Ethics and dissemination

For peer review only

Ethics approval was obtained in all participating countries. Results of surgical management of acute subdural hematoma and intracerebral hematoma/contusion will separately be submitted for publication in a peer-reviewed journal.

Trial registration

CENTER-TBI is registered within ClinicalTrials.gov with identifier NCT02210221 and Net-QuRe is registered with the Netherlands Trial Register NL 5761.

Article summary

Strengths and limitations of this study

- This comparative effectiveness study is a multicentre prospective observational cohort study that exploits variation in management strategies for intracranial hematomas to enable comparisons of the effectiveness of interventions.
- To overcome the bias by confounding the main analyses uses an instrumental variable approach; this approach is more robust to address unmeasured confounding than conventional individual patients level analysis methods
- Large sample sizes will be recruited: 1000 patients with acute subdural hematoma and 750 patients with intracerebral hematoma/contusion are expected, recruited in approximately 70 centres.
- Simulation studies confirmed the expected samples to be sufficient.
- The main limitation of this study is the absence of a randomised assignment of treatment strategy.

Keywords

Acute subdural hematoma, traumatic contusion, intracerebral hematoma, decompressive craniectomy.

For peer review only

Background

In Europe, over two million patients are admitted to hospital each year for traumatic brain injury (TBI), of whom 82.000 people die [1]. Survivors may have long-term physical, cognitive and mental disorders that often necessitate specialized care or rehabilitation programs. This debilitating morbidity has been estimated to lead to enormous societal costs [2]. An acute intracranial hematoma is the most frequently encountered pathological entity in TBI patients (Figure). Predominantly two specific subtypes, the acute subdural and an intracerebral hematoma or contusion (ASDH and t-ICH), occur in respectively 11 and 8% of all moderate TBI patients and up to 49% and 35% of all severe TBI patients [3-6]. Patients with an ASDH and/or t-ICH can show a wide array of symptoms, ranging from relatively mild complaints such as headache and nausea to severe conditions such as a comatose state (defined by a Glasgow Coma Scale (GCS) < 9).

For peer review only

Management of these traumatic hematomas can be challenging and requires the integration of clinical findings and diagnostic imaging. The main question is whether or not the patient needs to be immediately operated upon for evacuation of the hematoma and, secondly, if this surgical evacuation should be accompanied by a decompressive craniectomy (DC, i.e. leaving the bone flap out) or not. Among comatose patients with a large ASDH, direct evacuation of the hematoma leads to a lower mortality [3,4]. Although questioning the effectiveness of surgery in these patients has been compared to questioning the effectiveness of a parachute in skydiving [5,6], this cannot be generalized to most ASDH patients. Surgery may save a patient's life and preserve neurological function, some, however, may have an unsatisfactory functional outcome, ranging from severe neurological and cognitive deficits to a persistent vegetative state [7-9]. Furthermore, certain subgroups may not benefit from surgery because the damage by the primary injury is simply too devastating [9]. On the other hand, surgery may not always be necessary and a substantial proportion of patients managed conservatively have satisfactory outcomes [10-14]. In addition, timing of surgery plays a role, specifically for a t-ICH. Sometimes a t-ICH is initially managed conservatively, but may later be treated surgically when a patient deteriorates. The evacuation of the t-ICH can consist of removal of contused brain tissue. Finally, for the decision whether or not the evacuation of the hematoma should be accompanied by a DC, the surgeon weighs the increased complications rate of a DC against the risk of medically intractable diffuse brain swelling [15].

High quality evidence for these decisions (*if*, *when* and *how)* is not available. For all guidelines that relate to TBI, the Brain Trauma Foundation (BTF) guideline for surgical management of intracranial hematomas, devised in 2005 by an international panel of experts, is based solely on class III evidence [16,17]. As a result these complex decisions are often based on intuition, regional training and experience of the surgeon, leading to broad practice variation between centres, countries and even between surgeons within a centre [5,18-22]

Therefore, a systematic evaluation in a (prospective) comparative study is proposed with comprehensive assessment of outcome, including perceived quality of life. We consider an observational comparative effectiveness design the next best alternative to a randomised controlled trial (RCT). These clinical questions are difficult to address in a randomised trial due to several methodological, ethical and pragmatic concerns [23]. Most importantly, the hesitance of clinicians to randomise surgical treatments because of strong opinions on the best treatment hampers realising a RCT.

For peer review only

Here, we present the design of a pragmatic prospective observational cohort study of surgical strategies for ASDH and t-ICH, conducted in the Collaborative European NeuroTrauma Effectiveness Research in Traumatic Brain Injury (CENTER-TBI) study and the Dutch embedded complete chain of care Neurotraumatology Quality Registry (Net-QuRe) [24,25].

Objectives

The primary aim is to compare the effectiveness of acute surgery with expectant management in the treatment of 1) ASDH and 2) t-ICH. The secondary goal is 3) to assess the effectiveness of craniotomy compared to DC for ASDH and/or t-ICH.

For peer review only - http://bmjopen.bmj.com/site/about/guidelines.xhtml

Methods and design

Design

This study uses a comparative effectiveness research (CER) design, a multicentre prospective observational cohort study that exploits variation in neurotrauma care to create and compare parallel study groups. The multicentre design is necessary to ensure the required number of patients with different neurotrauma treatment strategies for ASDH and t-ICH. The study is conducted in neurosurgical trauma centres in Europe that participate in CENTER-TBI [24]. CENTER-TBI collects data of patients with clinical diagnosis of TBI and an indication for a CT scan [26]. Data for the cohort described in this protocol will partly be collected through CENTER-TBI. The Dutch centres not participating in CENTER-TBI will acquire data through Net-QuRe, in a separate database with a similar data collection protocol [25,27]. The research question and methodology described here were designed before the inclusion of patients.

Patient and public involvement

Patients were involved in the priority of the research questions and selection of outcome measures in a ‘patient advisory panel’ consisting of patients and their caregivers. Furthermore, some patients and their caregivers have been asked to join a focus group on the feasibility of the follow-up (burden of the follow-up, the design/length of the questionnaires) and to advice on other research questions. The patient panel and their caregivers will be informed about the developments of the study and will be invited to participate in research meetings and discussions. Also, patients and family are informed of the study results through dedicated websites (Center-tbi.eu and Net-QuRe.nl).

A public debate is going on in the Netherlands about whether or not patients are treated too much at an older age or end-of-life stage. This debate has led to the start of formal campaign ‘Choosing Wisely’ of which the senior author WPE is organizing member [28]. The appropriateness of surgical TBI treatment has been prioritized.

Eligible study patients

The patients are selected from the observational cohorts from CENTER-TBI or Net-QuRe. Patients presenting to the emergency room with a clinical and radiological diagnosis of ASDH and/or a t-ICH

For peer review only

For peer review only - http://bmjopen.bmj.com/site/about/guidelines.xhtml

are eligible for inclusion.

The in- and exclusion criteria are as following.

Inclusion criteria:

- ASDH and/or a large (>10 cc) t-ICH on a CT-scan
- Acute presentation (< 24 hours of injury) with history of head trauma
- Clinical indication for admission (ward or ICU)

Exclusion criteria:

- An ASDH or t-ICH due to penetrating injury, a spontaneous or iatrogenic ASDH/t-ICH
- Severe pre-existing neurological disorder that would confound outcome assessments

For peer review only

Patients are not excluded based on other clinical and radiological characteristics (such as: advanced age, antiplatelet/anticoagulant use, small hematoma volume). Radiological criteria for an ASDH or t-ICH/contusion are a high-density lesion, with or without radiological signs of raised intracranial pressure (ICP) and with or without mass effect (i.e. midline shift, compression of ventricles and/or basal systems). The minimal size of the t-ICH needs to be 10cc to be included.

<u>Intervention procedures</u>

Inherent to the observational design of this study the management strategies under investigation proceed according to local emergency- and intensive care protocols or surgeon's expertise. Consequently, the resulting variation in management is accepted and analysed. To gain insight into this variation detailed information is collected on the reasons for specific interventions or management strategies (see section 'why' questions).

Surgical strategy

Surgical treatment consists of evacuation of the hematoma and/or contusion with a craniotomy, or with a DC, defined as hematoma evacuation plus leaving a large portion of the skull open to allow brain swelling in the secondary phase, preventing subsequent brain injury. Generally, in Europe a craniotomy is performed for hematoma evacuation and DC when (intractable) swelling is seen intraoperatively or when swelling is expected (preventive). The decision for a DC can be made primarily, or secondarily by increasing the defect of the bone flap that is formed during a normal

For peer review only - http://bmjopen.bmj.com/site/about/guidelines.xhtml

craniotomy. In conjunction to these surgical procedures the neurosurgeon will decide to place an intracranial pressure (ICP) monitor or not. The ICP device can be an intraparenchymal sensor or an external ventricular drain with a transducer for the ICP. The latter has an option to drain cerebrospinal fluid (CSF) and thereby lower ICP.

The operation will be performed by a qualified neurosurgeon or neurosurgical resident. The techniques for evacuating such hematomas are well established, although specific components of the operations may differ between surgeons. Our aim will be to collect pertinent operative data on a standardized data collection form.

The postoperative care on the ward or intensive care unit (ICU) generally is protocolized in European centres. The length of hospital stay will differ considerably between patients, ranging from one day to several months, mainly related to the severity of the injury. The aim for a patient is to be discharged as soon as possible. Furthermore, the supportive care is provided as described in the section 'expectant management'.

Expectant management, with possibly delayed surgery

For patients admitted to the ward, monitoring is in general by clinical neurological control (GCS and motor strength) with or without CT brain follow-up, whereas for those patients admitted to the ICU (mostly severe TBI) the diagnostic and therapeutic options include ICP monitoring with medical management of intracranial hypertension (i.e. hyperosmolar therapies, hyperventilation etc) and cerebral perfusion pressure (CPP).

Follow-up CT scans, performed during hospitalization, are collected and analysed centrally, independent from the treating physicians. Hereby, an estimation is made about the proportion of the evacuated hematoma and the change in density of lesions.

Patients will be allocated to one of the treatment arms based on the initial treatment strategy. The data collection includes questions after each CT that ask whether or not the patient is transferred to the OR for an operative procedure. In doing so, the treatment 'arms' in this study can be carefully controlled based on the first CT on presentation (showing an ASDH and/or a t-ICH). The initial treatment regimen chosen will be one of either treatment arms, analysed according to an intention-to-treat approach.

For peer review only

For peer review only - http://bmjopen.bmj.com/site/about/guidelines.xhtml

Data collection

Data of care management by hospital personnel are registered in all departments. Data collection is done in a standardized electronic database, based on the 'common data elements' for TBI and web-based data collection protocol [29,30].

Practice variation and provider profiling

Parallel to a survey on regional practice variation towards ASDH management in the Netherlands, we have performed provider profiling of neurosurgical care for TBI in Europe [31]. This study provides an exploration of the organization of neurosurgical care and treatment policy of TBI in all study centres. Such centre characterization allows specification of the local policy and standardized protocol. Structures and processes of care to be studied are patient volume, location of first ER evaluation, level of the trauma centre, referral policy, number of neurosurgeons, type of ICU and 24/7 CT availability. In addition, with regard to post-acute care, routine follow-up for ER patients, ICU approaches to fluid load, hyperventilation, hyperosmolar therapy, timing of intracranial surgery, timing of extracranial surgery, glucose management, cerebral spinal fluid (CSF) drainage, DC, CPP management, coagulopathy treatment and ICP monitoring (parenchymal or CSF catheter). Whether or not consensus for divergent clinical decisions is agreed upon and whether or not hospital protocols are available and applied.

Admission data

In short, the 'common data elements' entail the collection of patient characteristics including demographics, comorbidities, associated extracranial injuries, neurological condition, prehospital information, hypoxic and hypotensive periods, and CT abnormalities. For the purpose of CER detailed information on processes of care will be collected within CENTER-TBI and Net-QuRe, including timing (of first CT, second CT, operation), surgery parameters, pre-hospital management and IC therapies (including ICP: pharmacological and/or DC). These data are collected on patient level, but also on hospital level (see previous paragraph).

For peer review only

'Why' questions

The observational design poses a challenge for inferring that the surgery caused the outcome instead of other factors. Several known and measured confounders (pupillary abnormalities, GCS and hematoma characteristics on CT) can be accounted for. However, for the decision to operate or not in intracranial hematoma, neurosurgeons select certain clinical, radiological and subjective 'gut feeling' characteristics that would normally go unmeasured. Therefore, the following efforts are undertaken to collect variables that normally will go unmeasured. To assess the effectiveness of different treatments for ASDH and t-ICH additional data are collected on an individual (doctor) level; the neurosurgeon, ICU physician and/or neurologist is asked to give their indication/reason to choose for surgery or conservative management (e.g. hematoma size, mass effect, clinical symptoms, clinical deterioration and/or other motivation), his or her motivation for the chosen procedure (DC or craniotomy) and the anticipated prognosis of the patient.

The motivation for surgery and the prognosis is collected before the decision for surgery or conservative management because afterwards the motivation could have been changed due to several factors such as intraoperative findings and the clinical course of the patient after the surgery. Because of the infrastructure in TBI care, in which the clinician on-call is often outside the hospital and - in case of the neurosurgeon - will start with the surgery right after arrival in the hospital, the neurological and neurosurgical residents will assess their supervising clinician's motivation before the decision is carried out. To control this process, the date and time of collecting these variables is collected as well.

For peer review only

Outcome measurements and endpoints

Within CENTER-TBI and Net-QuRe the outcome measures are assessed by face-to-face interviews, postal or emailed questionnaires or by telephone interviews at 3, 6 and 12 months after injury. Outcome assessment is done naïve for the research questions.

The primary endpoint is the 6-month Glasgow Outcome Score - Extended (GOSE, [32]). The Glasgow Outcome Scale Extended (GOSE) is the most commonly used outcome measure in TBI. The GOSE grades disability on an 8-point scale incorporating physical deficits as well as emotional

For peer review only - http://bmjopen.bmj.com/site/about/guidelines.xhtml

and cognitive disturbances affecting disability [33,34]. The GOSE is designed as a structured interview and can be applied through telephone [35] and the mail [36].

Secondary endpoints are mortality, structural hematoma changes on CT, frequency and type of neurosurgical interventions, ICU and hospital length of stay (days), complications (hydrocephalus, intracranial haemorrhage, infection, pulmonary embolism, deep vein thrombosis and death), 'treatment failure' during the initial hospital admittance (patient in the expectant group who are operated at a delayed moment or patients in the early surgery group who are operated again), discharge to home (from the hospital, rehabilitation facility or nursing home), quality of life 6 and 12 months postinjury with the SF-12, the brain injury specific Quality of Life after Brain Injury questionnaire (Qolibri) [37] and cognitive tests. Details of these and other outcome measures are provided in our previous publication [24].

For peer review only

Data analysis

Patient characteristics, hospital characteristics, and variation in both treatment and outcome variation will be described using descriptive statistics. To assess differences between groups, appropriate tests will be employed according to distribution and scale of measurement (Student's t-tests or Mann-Whitney U tests for continuous variables and Chi-squared tests, or Fisher's exact test for categorical variables). To examine effectiveness of interventions, proportional odds logistic regression models with the 8-point ordinal GOSE as outcome variable will be used. A proportional odds model increases statistical power in comparison to a conventional logistic regression model with a binary outcome [38]. The odds ratios derived from a proportional odds regression model could be interpreted as the odds ratio (OR) for shifting over the GOSE [38].

The main challenge in the analyses is how to estimate a treatment effect in these observational data with strong confounding. Conventional methods, patient-level analyses with covariate adjustment in regression modelling and propensity score matching, can insufficiently account for the (unmeasured) confounding in TBI [39]. Therefore, the main analyses will use the between-hospital variation in treatment for determining effectiveness by comparing regional treatment strategies. This is an instrumental variable (IV) approach. The instrument is the proportion of patients exposed to the

For peer review only - http://bmjopen.bmj.com/site/about/guidelines.xhtml

intervention per hospital, determined as the proportion initially operated ASDH of total ASDH patients, initially operated t-ICH of total t-ICH patients and proportion of total ASDH or t-ICH patients exposed to primary DC (that forms a proxy for 'aggressiveness' of the neurosurgical staff). Total ASDH and ICH numbers are available through the registry. The instrument is entered as an independent variable to the analyses. The unmeasured and measured confounding at the hospital level, for example hospitals that perform more surgery also more often perform other treatments, is overcome with a multilevel analysis model [40]. In this model the random intercept should capture the measured and unmeasured confounders at hospital level, resulting in unbiased treatment effect estimates. The random intercept for each hospital represents the unexplained hospital effect (beyond all factors included in the model, including the instrument treatment preference). Assumptions of the IV approach will be checked according to our previous published case study [39].

For these analyses, hospitals contributing at least 15 patients to the study sample are included to minimize the influence of chance. To increase statistical power, adjustment for potential patient level confounding will be made by adding the strongest predictors of outcome (age, GCS, pupillary response, CT characteristics (hematoma thickness and volume, subarachnoid haemorrhage, basal cistern compromise, other focal or diffuse lesions), hypoxic or hypotensive episodes and extracranial injuries) as covariates [41]. These factors were determined by prognostic modelling in the International Mission for Prognosis and Analysis of Clinical Trials (IMPACT) study based on a dataset of ten RCTs and three observational studies [42]. Thus, the treatment effect parameter will be the estimate for the effect of 'aggressiveness' on outcome from a random effects ordinal regression model with hospital as a random intercept.

In sensitivity analyses the instrument validity will be further explored by quantifying a priori collected data, the results of our survey [18] and the provider profiling of CENTER-TBI [19], and comparing these to the post-hoc derived relative proportion exposed to the intervention per hospital.

In the secondary analyses, conventional regression modelling with covariate adjustment and propensity score matching is performed. In both these analyses actual treatment will be a binary treatment variable and GOSE as ordinal outcome variable. For ASDH surgery effectiveness,

For peer review only

confounding will be controlled for by adding age, GCS, pupil reactivity, hematoma thickness and midline shift as covariates in the model. For t-ICH effectiveness, confounding adjustment for age, GCS, hematoma volume, pupil reactivity, hematoma laterality and midline shift. For DC effectiveness, by age, GCS, pupil reactivity, midline shift and hematoma size (ASDH: thickness; t-ICH: volume).

Importantly, the effect of surgery is probably not uniform, as is suggested by empirical evidence [43,44] and by clinical experience. Therefore, effect modification by the following variables will be tested using interaction terms: GCS of ≥ 9, hematoma size >10 mm in diameter, midline shift >5 mm, time to treatment and baseline prognostic risk [32].

<u>Sample size</u>

In CENTER-TBI and Net-QuRe together approximately 1000 patients with ASDH are expected. For t-ICH, 750 patients are expected. Parallel to the core study, 3500 and 3000 ASDH and ICH patients are expected in the registry respectively. These patients are recruited in approximately 70 centres. Standard sample size calculations for these specific analyses are not readily available. Therefore, a simulation study was performed to calculate statistical power. The assumptions for these calculations were the following: 30, 50 or 70 hospitals, variation of intracranial operation among hospitals 10 to 90% and an effect estimate of OR 0.6 on unfavourable outcome. In addition, we assumed covariate adjustment and the ordinal analysis to increase power with 40 to 49% [38]. The simulation confirmed these sample sizes to be sufficient to obtain a power of 80% to detect a difference (assuming a 2-sided significance 0.05).

This is in line with preparatory simulation study we performed in which a true treatment effect was simulated specifically to assess the TBI specific associations between covariates and outcome [39]. This simulation study was built around the International and North American Tirilazad trial dataset (86 hospitals between 1992 and 1994) of the IMPACT dataset [42], which was inflated to CENTER-TBI/Net-QuRe numbers (respectively 750 and 1000 patients from 70 hospitals). We simulated a hypothetical intervention with an OR 1.5. For the association between the hypothetical intervention and confounders, we used the observed associations between intracranial operation and confounders in the Tirilazad dataset. We used six-month binary (functional) outcome as dependent variable, which

For peer review only

was generated based on a combination of the prognostic effect of the confounders and the effect of the hypothetical intervention.

For analyses and simulations R statistical software with add-ons (the *rms* and *lme4* packages) is used.

Missing data

Missing baseline data will be imputed with multiple imputation (n=5).

Reporting

Reporting of our study will follow the STROBE statement with a special focus on instrumental variable analyses recommendations [45]

Study limitations

The main limitation of this study is the absence of a randomised assignment of treatment or strategy. Risks of confounding by indication are reduced by instrumental variable analysis. The success of the primary analysis however, depends on the strength of the instrument, i.e. the difference in aggressive versus conservative practice style between physicians during the study period. The results of the provider profiling (before the study) are encouraging. As secondary analyses we perform more conventional approaches to adjust for confounding by indication (multivariable adjustment and propensity scores). The results of analytical approaches will be interpreted in the light of the assumptions they require and to what extent these are likely to be fulfilled in the data. The final conclusion will be drawn based on the joint results of all analyses.

For peer review only

Discussion

There is controversy with regard to the initial neurosurgical management of ASDH and t-ICH. First, neurosurgeons are faced with an acute decision to operate or not, and second, are confronted by the choice to evacuate the hematoma with or without a DC. The complexity lies in the balance between too liberal surgical indications with an increased number of survivors with severe disabilities against inappropriate conservative management with unnecessary death and disability. In combination with the circumstances, i.e. urgency and time pressure as well as absence of peer consultation, these treatment decisions have been shown to lead to variation in surgical treatment between surgeons.

The proposed study will provide a strong level of evidence for surgical management of ASDH and t-ICH. We expect that the large natural existing practice variation in management of these intracranial lesions [18,19] could in part explain the unexplained between-centre variability in outcome in TBI [46]. Thereby, the impact on patients will probably be significant. Recognizing and implementing the most effective clinical treatment strategy could be an important step towards reducing the widely differing injury mortality rates across Europe [47].

Current and ongoing studies are sparse. Since the BTF guideline, which was based on merely retrospective studies with small or selected study populations that were performed more than 10 years before the guideline [5,10,11,48], there have been only some comparative studies. In our own retrospective analyses, early ASDH evacuation might be associated with lower odds of mortality and unfavourable outcome (GOS $\leqq$ 3) [43]. This is the first report showing an effect estimate of surgery for ASDH. The clinical effectiveness of an early evacuation for t-ICH was challenged in the Surgical Trial in Traumatic Intracerebral Haemorrhage (STITCH-Trauma), an international multicentre pragmatic randomised controlled trial [49]. The study started in October 2009 but was halted due to a disbalance in recruited patients per country. In the analysis of the included patients, a strong (but non-significant) tendency towards benefit of early surgery was found on the primary endpoint the dichotomous GOS and there were significantly more deaths in the initial conservative treatment group. The effectiveness of a primary DC in patients with ASDH is currently being assessed in the recruiting RESCUE-ASDH randomised trial [15].

For peer review only - http://bmjopen.bmj.com/site/about/guidelines.xhtml

Thus, these traumatic hematomas confront the neurosurgeon with a challenging surgical decision-making task, which, most likely due to a lack of general evidence, leads to broad variation in current surgical practice patterns. While RCTs can be delivered and provide high level evidence, they are challenging to conduct, hence the rationale for other methodological intervention paradigms. The high-quality observational study presented in this article, with a focus on the analysis of the differences in management and outcome, is expected to provide the much-needed further evidence in the field of surgical management of traumatic focal lesions.

For peer review only

For peer review only - http://bmjopen.bmj.com/site/about/guidelines.xhtml

Abbreviations

ASDH: acute subdural hematoma,

CENTER-TBI: Collaborative European NeuroTrauma Effectiveness research in TBI

CER: comparative effectiveness research,

CPP: cerebral perfusion pressure,

CSF: cerebrospinal fluid,

CT: computed tomography,

ER: emergency room,

GCS: Glasgow Coma Score,

GOSE: Glasgow Outcome Scale Extended,

ICU: intensive care unit,

ICP: intracranial pressure,

IMPACT: International Mission for Prognosis and Analysis of Clinical Trials in TBI,

IV: instrumental variable,

Net-QuRe: Neurotraumatology Quality Registry,

OR: operating room,

Qolibri: Quality of Live in Brain Injury,

RCT: randomised clinical trial,

TBI: traumatic brain injury,

t-ICH: intracerebral hematoma/contusion.

For peer review only

Declarations

Ethics and dissemination

Ethical approval for the studies CENTER-TBI and Net-QuRe has been received from each participating centre in the study before recruitment started. The list of sites, Ethical Committees, approval numbers and approval dates can be found on the website: https://www.center-tbi.eu/project/ethical-approval. Written informed consent is obtained from the patient or the legal first representative on the ER and preferably as soon as possible. Center-TBI is registered at ClinicalTrials.gov (NCT02210221) and Net-QuRe is registered at the Netherlands Trial Register (NL 5761) [26,27].

Results of surgical management of ASDH and t-ICH will separately be submitted for publication in a peer-reviewed journal. The completion dates of CENTER-TBI and Net-QuRe are April 1st, 2020 and July 1st, 2022 respectively.

The data that support the findings of this study are available but restrictions apply to the availability of these data, which were used under license for the current study, and so are not publicly available. Data are however available from the authors upon reasonable request, with permission of the CENTER-TBI and Net-QuRe management teams.

Competing interests

The authors declare that they have no competing interests.

Funding

The project described in this study protocol is funded by the *Hersenstichting Nederland* (the Dutch Brain Foundation, grant number ps2014.06) for Net-QuRe and the European Union seventh Framework Program (grant 602150) for CENTER-TBI. Additional support for CENTER-TBI was obtained from the Hannelore Kohl Stiftung (Germany), from OneMind (USA), from Integra LifeSciences Corporation (USA), and from NeuroTrauma Sciences (USA). The funding bodies had no role in the design of the study, collection, analysis, and interpretation of data and in writing the manuscript. This research received no specific grant from any funding agency in the public, commercial or not-for-profit sectors.

For peer review only

Authors PH and AK are supported by the NIHR Global Health Research Group on Neurotrauma, which was commissioned by the National Institute for Health Research (NIHR) using UK aid from the UK Government (project 16/137/105). The views expressed in this publication are those of the author(s) and not necessarily those of the NIHR or the Department of Health and Social Care. PH is supported by a Research Professorship from the NIHR, the NIHR Cambridge Biomedical Research Centre, and the Royal College of Surgeons of England. AK is supported by the Royal College of Surgeons of England and a Clinical Lectureship, School of Clinical Medicine, University of Cambridge

Author contributions

TvE conceived of the study design, performed the statistical analysis and wrote the manuscript. GdR, VV, MC, LP, HL, BD, MH, GR, DM, PH, AK, ES, AM and WP participated in the design of the study and helped to draft the manuscript. DN, IC, HL and TvE performed the simulation studies. All authors read and approved the final manuscript.

Acknowledgements

Not applicable

For peer review only

References

1 Majdan M, Plancikova D, Brazinova A, *et al.* Epidemiology of traumatic brain injuries in Europe: a cross-sectional analysis. *Lancet Public Health* 2016;**1**:e76–e83. doi:10.1016/S2468-2667(16)30017-2

2 Faul M, Wald MM, Rutland-Brown W, *et al.* Using a Cost-Benefit Analysis to Estimate Outcomes of a Clinical Treatment Guideline: Testing the Brain Trauma Foundation Guidelines for the Treatment of Severe Traumatic Brain Injury. *The Journal of Trauma: Injury, Infection, and Critical Care* 2007;**63**:1271–8. doi:10.1097/TA.0b013e3181493080

3 Seelig JM, Becker DP, Miller JD, *et al.* Traumatic acute subdural hematoma: major mortality reduction in comatose patients treated within four hours. *N Engl J Med* 1981;**304**:1511–8. doi:10.1056/NEJM198106183042503

4 Bullock MR, Chesnut R, Ghajar J, *et al.* Surgical management of acute subdural hematomas. *Neurosurgery* 2006;**58**:S16–24–discussionSi–iv.

5 Compagnone C, Murray GD, Teasdale GM, *et al.* The Management of Patients with Intradural Post-Traumatic Mass Lesions: A Multicenter Survey of Current Approaches to Surgical Management in 729 Patients Coordinated by the European Brain Injury Consortium. *Neurosurgery* 2005;:1183–92. doi:10.1227/01.NEU.0000186239.10915.09

6 Smith GCS, Pell JP. Parachute use to prevent death and major trauma related to gravitational challenge: systematic review of randomised controlled trials. *BMJ* 2003;**327**:1459–61.

7 Tallon JM, Ackroyd-Stolarz S, Karim SA, *et al.* The epidemiology of surgically treated acute subdural and epidural hematomas in patients with head injuries: a population-based study. *Can J Surg* 2008;**51**:339–45.

8 Li LM, Kolias AG, Guilfoyle MR, *et al.* Outcome following evacuation of acute subdural haematomas: a comparison of craniotomy with decompressive craniectomy. *Acta Neurochir* 2012;**154**:1555–61. doi:10.1007/s00701-012-1428-8

9 Nijboer JMM, van der Naalt J, Duis ten H-J. Patients beyond salvation? Various categories of trauma patients with a minimal Glasgow Coma Score. *Injury* 2010;**41**:52–7. doi:10.1016/j.injury.2009.05.030

10 Dent DL, Croce MA, Menke PG, *et al.* Prognostic factors after acute subdural hematoma. *The Journal of Trauma: Injury, Infection, and Critical Care* 1995;**39**:36–42–discussion42–3.

11 Mathew P, Oluoch-Olunya DL, Condon BR, *et al.* Acute subdural haematoma in the conscious patient: outcome with initial non-operative management. *Acta Neurochir* 1993;**121**:100–8.

12 Bus S, Verbaan D, Kerklaan BJ, *et al.* Do older patients with acute or subacute subdural hematoma benefit from surgery? *Br J Neurosurg* 2019;**33**:51–7. doi:10.1080/02688697.2018.1522418

13 Gernsback JE, Kolcun JPG, Richardson AM, *et al.* Patientem Fortuna Adiuvat: The Delayed Treatment of Surgical Acute Subdural Hematomas-A Case Series. *World Neurosurg* 2018;**120**:e414–20. doi:10.1016/j.wneu.2018.08.095

14 Servadei F, Nasi MT, Cremonini AM, *et al.* Importance of a reliable admission Glasgow Coma Scale score for determining the need for evacuation of posttraumatic subdural hematomas: a prospective study of 65 patients. *The Journal of Trauma: Injury, Infection, and Critical Care* 1998;**44**:868–73.

For peer review only - http://bmjopen.bmj.com/site/about/guidelines.xhtml

15 Kolias AG, Viaroli E, Rubiano AM, *et al.* The Current Status of Decompressive Craniectomy in Traumatic Brain Injury. *Current Trauma Reports* 2018;:1–7. doi:10.1007/s40719-018-0147-x

16 Bullock MR, Chesnut R, Ghajar J, *et al.* Surgical management of acute subdural hematomas. *Neurosurgery* 2006;**58**:S16–24–discussionSi–iv.

17 Bullock MR, Chesnut R, Ghajar J, *et al.* Surgical management of traumatic parenchymal lesions. *Neurosurgery* 2006;**58**:S25–46–discussionSi–iv.

18 van Essen TA, de Ruiter GCW, Kho KH, *et al.* Neurosurgical Treatment Variation of Traumatic Brain Injury: Evaluation of Acute Subdural Hematoma Management in Belgium and The Netherlands. *Journal of Neurotrauma* 2017;**34**:881–9. doi:10.1089/neu.2016.4495

19 Essen TA, Boogert HF, Cnossen MC, *et al.* Variation in neurosurgical management of traumatic brain injury: a survey in 68 centers participating in the CENTER-TBI study. 2018;:1–15. doi:10.1007/s00701-018-3761-z

20 Kolias AG, Scotton WJ, Belli A, *et al.* Surgical management of acute subdural haematomas: current practice patterns in the United Kingdom and the Republic of Ireland. *Br J Neurosurg* 2013;**27**:330–3. doi:10.3109/02688697.2013.779365

21 Ghajar J, Hariri RJ, Narayan RK, *et al.* Survey of critical care management of comatose, head-injured patients in the United States. *Crit Care Med* 1995;**23**:560–7.

22 Murray GD, Teasdale GM, Braakman R, *et al.* The European Brain Injury Consortium survey of head injuries. *Acta Neurochir* 1999;**141**:223–36.

23 Stein SC. Comparative effectiveness in neurosurgery: what it means, how it is measured, and why it matters. *Neurosurgical FOCUS* 2012;**33**:E1. doi:10.3171/2012.2.FOCUS1232

24 Maas AIR, Menon DK, Steyerberg EW, *et al.* Collaborative European NeuroTrauma Effectiveness Research in Traumatic Brain Injury (CENTER-TBI): a prospective longitudinal observational study. *Neurosurgery* 2015;**76**:67–80.

25 Van Essen TA, Peppel L, Van Dijck J, Dijkman M, Hester F. Lingsma, et al. The Net-QuRe study: an observational cohort Study on neurosurgical and rehabilitation strategies for traumatic brain injury. The 3rd Joint Symposium of the International and National Neurotrauma Societies and AANS/CNS Section on Neurotrauma and Critical Care August 11–16, 2018 Toronto, Canada. *Journal of Neurotrauma* 2018;**35**:A–1–A–285. doi:10.1089/neu.2018.29013.abstracts

26 Maas AIR. CENTER-TBI: Collaborative European NeuroTrauma Effectiveness Research in TBI. *clinicaltrialsgov* 2014.

27 Nederlands Trial Register [Internet]. Amsterdam: Academic Medical Center (The Netherlands). 2004 Oct 26. Identifier Trial NL5761; 2016 July 28. Available from: https://www.trialregister.nl/trial/5761.

28 Choosing Wisely Netherlands Campaign. https://www.demedischspecialist.nl/onderwerp/campagne-verstandig-kiezen.

29 Maas AIR, Harrison-Felix CL, Menon D, *et al.* Standardizing data collection in traumatic brain injury. *Journal of Neurotrauma* 2011;**28**:177–87. doi:10.1089/neu.2010.1617

30 Maas AI, Harrison-Felix CL, Menon D, *et al.* Common data elements for traumatic brain injury: recommendations from the interagency working group on demographics and

For peer review only

clinical assessment. *Arch Phys Med Rehabil* 2010;**91**:1641–9. doi:10.1016/j.apmr.2010.07.232

31 van Essen TA, Boogert den HF, Cnossen MC, *et al.* Variation in neurosurgical management of traumatic brain injury: a survey in 68 centers participating in the CENTER-TBI study. *Acta Neurochir* Published Online First: December 2018. doi:10.1007/s00701-018-3761-z

32 Wilson JT, Pettigrew LE, Teasdale GM. Structured interviews for the Glasgow Outcome Scale and the extended Glasgow Outcome Scale: guidelines for their use. *Journal of Neurotrauma* 1998;**15**:573–85.

33 Shukla D, Devi BI, Agrawal A. Outcome measures for traumatic brain injury. *Clin Neurol Neurosurg* 2011;**113**:435–41. doi:10.1016/j.clineuro.2011.02.013

34 Bullock MR, Merchant RE, Choi SC, *et al.* Outcome measures for clinical trials in neurotrauma. *Neurosurgical FOCUS* 2002;**13**:ECP1.

35 Pettigrew LEL, Wilson JTL, Teasdale GM. Reliability of ratings on the Glasgow Outcome Scales from in-person and telephone structured interviews. *Journal of Head Trauma Rehabilitation* 2003;**18**:252–8.

36 Wilson JTL, Edwards P, Fiddes H, *et al.* Reliability of postal questionnaires for the Glasgow Outcome Scale. *Journal of Neurotrauma* 2002;**19**:999–1005. doi:10.1089/089771502760341910

37 Steinbuechel von N, Petersen C, Bullinger M. Assessment of health-related quality of life in persons after traumatic brain injury--development of the Qolibri, a specific measure. *Acta Neurochir Suppl* 2005;**93**:43–9.

38 Roozenbeek B, Lingsma HF, Perel P, *et al.* The added value of ordinal analysis in clinical trials: an example in traumatic brain injury. *Critical Care* 2011;**15**:R127. doi:10.1186/cc10240

39 Cnossen MC, van Essen TA, Ceyisakar IE, *et al.* Adjusting for confounding by indication in observational studies: a case study in traumatic brain injury. *Clinical Epidemiology* 2018;**10**:841–52.

40 Li B, Lingsma HF, Steyerberg EW, *et al.* Logistic random effects regression models: a comparison of statistical packages for binary and ordinal outcomes. *BMC Med Res Methodol* 2011;**11**:77. doi:10.1186/1471-2288-11-77

41 Hernandez AV, Steyerberg EW, Butcher I, *et al.* Adjustment for strong predictors of outcome in traumatic brain injury trials: 25% reduction in sample size requirements in the IMPACT study. *Journal of Neurotrauma* 2006;**23**:1295–303. doi:10.1089/neu.2006.23.1295

42 Lingsma H, Andriessen TMJC, Haitsema I, *et al.* Prognosis in moderate and severe traumatic brain injury: external validation of the IMPACT models and the role of extracranial injuries. *J Trauma Acute Care Surg* 2013;**74**:639–46.

43 Van Essen TA, Dijkman M, Cnossen MC, *et al.* Comparative effectiveness of surgery for traumatic acute subdural hematoma in an aging population. *Journal of Neurotrauma* Published Online First: September 2018. doi:10.1089/neu.2018.5869

44 Gregson BA, Mitchell P, Mendelow AD. Surgical Decision Making in Brain Hemorrhage. *Stroke* 2019;**50**:1108–15. doi:10.1161/STROKEAHA.118.022694

For peer review only

For peer review only - http://bmjopen.bmj.com/site/about/guidelines.xhtml

45 Davies NM, Smith GD, Windmeijer F, *et al.* Issues in the reporting and conduct of instrumental variable studies: a systematic review. *Epidemiology* 2013;**24**:363–9. doi:10.1097/EDE.0b013e31828abafb

46 Lingsma HF, Roozenbeek B, Li B, *et al.* Large Between-Center Differences in Outcome After Moderate and Severe Traumatic Brain Injury in the International Mission on Prognosis and Clinical Trial Design in Traumatic Brain Injury (IMPACT) Study. *Neurosurgery* 2011;**68**:601–8. doi:10.1227/NEU.0b013e318209333b

47 Eurosafe. Injuries in the European Union: Summary of Injury Statistics for the Years 2008-2010. Amsterdam, The Netherlands: European Association for Injury Prevention and Safety Promotion. Available at: http://ec.europa.eu/health/data_collection/docs/idb_report_2013_en.pdf. 2013.

48 Servadei F, Nasi MT, Cremonini AM, *et al.* Importance of a reliable admission Glasgow Coma Scale score for determining the need for evacuation of posttraumatic subdural hematomas: a prospective study of 65 patients. *The Journal of Trauma: Injury, Infection, and Critical Care* 1998;**44**:868–73.

49 Mendelow AD, Gregson BA, Rowan EN, *et al.* Early surgery versus initial conservative treatment in patients with traumatic intracerebral haemorrhage [STITCH(Trauma)]: the first randomised trial. *Journal of Neurotrauma* Published Online First: March 2015. doi:10.1089/neu.2014.3644

50 Maas AIR, Menon DK, Adelson PD, *et al.* Traumatic brain injury: integrated approaches to improve prevention, clinical care, and research. *The Lancet Neurology* 2017;**16**:987–1048. doi:10.1016/S1474-4422(17)30371-X

For peer review only

Figure legend

Figure: Different types of post-traumatic intracranial haematoma

(A) Epidural haematoma: a collection of blood between the skull and the outer membrane covering the brain (dura mater). (B) Subdural haematoma: a collection of blood located underneath the dura mater, generally associated with bruising of the underlying brain tissue (contusions). (C) Haemorrhagic contusion and intracerebral haematoma: lesions that reflect similar underlying pathologies, ranging from local bruising (contusions) to bleeding into the brain tissue (haematoma). Figure courtesy of Maartje Kunen, Medical Visuals, Arnhem, Netherlands. Reproduced with permission from [50].

For peer review only

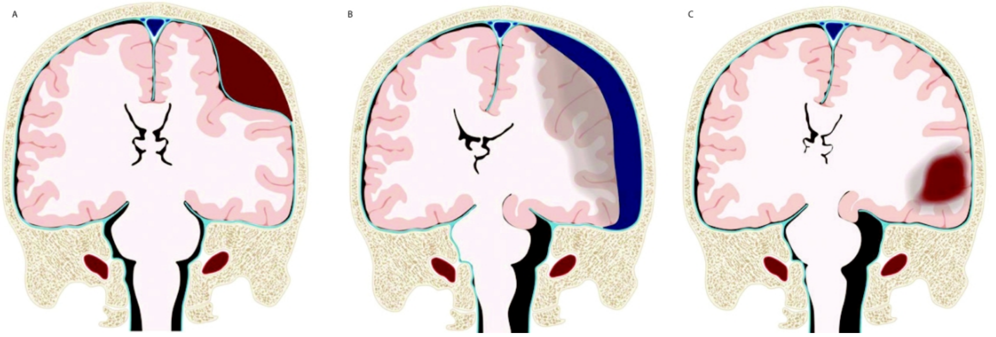

Figure: Different types of post-traumatic intracranial haematoma
(A) Epidural haematoma: a collection of blood between the skull and the outer membrane covering the brain (dura mater). (B) Subdural haematoma: a collection of blood located underneath the dura mater, generally associated with bruising of the underlying brain tissue (contusions). (C) Haemorrhagic contusion and intracerebral haematoma: lesions that reflect similar underlying pathologies, ranging from local bruising (contusions) to bleeding into the brain tissue (haematoma). Figure courtesy of Maartje Kunen, Medical Visuals, Arnhem, Netherlands. Reproduced with permission from [50].

448x149mm (300 x 300 DPI)
